# Application of Plasmid Engineering to Enhance Yield and Quality of Plasmid for Vaccine and Gene Therapy

**DOI:** 10.3390/bioengineering6020054

**Published:** 2019-06-19

**Authors:** Olusegun Folarin, Darren Nesbeth, John M. Ward, Eli Keshavarz-Moore

**Affiliations:** Advanced Center for Biochemical Engineering, University College London, London WC1E 6BT, UK; olusegun.folarin.14@ucl.ac.uk (O.F.); d.nesbeth@ucl.ac.uk (D.N.); j.ward@ucl.ac.uk (J.M.W.)

**Keywords:** *E. coli*, plasmid bioprocessing, plasmid supercoiling, superhelical density, plasmid engineering, nanoparticle tracking analysis (NTA)

## Abstract

There is an increased interest in plasmid DNA as therapeutics. This is evident in the number of ongoing clinical trials involving the use of plasmid DNA. In order to be an effective therapeutic, high yield and high level of supercoiling are required. From the bioprocessing point of view, the supercoiling level potentially has an impact on the ease of downstream processing. We approached meeting these requirements through plasmid engineering. A 7.2 kb plasmid was developed by the insertion of a bacteriophage Mu strong gyrase-binding sequence (Mu-SGS) to a 6.8 kb pSVβ-Gal and it was used to transform four different *E. coli* strains, and cultured in order to investigate the Mu-SGS effect and dependence on strain. There was an increase of over 20% in the total plasmid yield with pSVβ-Gal398 in two of the strains. The supercoiled topoisomer content was increased by 5% in both strains leading to a 27% increase in the overall yield. The extent of supercoiling was examined using superhelical density (σ) quantification with pSVβ-Gal398 maintaining a superhelical density of −0.022, and pSVβ-Gal −0.019, in both strains. This study has shown that plasmid modification with the Mu-phage SGS sequence has a beneficial effect on improving not only the yield of total plasmid but also the supercoiled topoisomer content of therapeutic plasmid DNA during bioprocessing.

## 1. Introduction

The past few decades have seen a rise in the focus of plasmid DNA for biotherapeutic production, with applications including vaccines and gene therapy. The reasons include, but not exhaustively, the ability to elicit both humoral and cell-mediated immune response [1,2,3] as well as the possibility of having multiple protection from one preparation. The rise in the application of plasmid DNA for vaccine and gene therapy is evident in ongoing clinical trials, with about 17.4% of all gene therapy clinical trials involving plasmid DNA [4]. Although there are presently no approved DNA vaccines for use in humans, there has been tremendous progress in the development of these vaccines, particularly DNA vaccines targeting HPV-derived onco-antigens [5], and a number of completed clinical trials on DNA vaccines for oncological indications which are extensively discussed in [6,7]. It is therefore no surprise that this particular class of biomolecule is receiving such great attention.

An ample amount of plasmid DNA (pDNA) is required (in mg) because not all of it is integrated during transfection and it must be mostly in the supercoiled form (in comparison to a linear and nicked form) to be effective for biotherapeutic applications [8]. Therefore, two bioprocess challenges that need to be addressed are the yield and the quality (i.e., maintaining a high supercoiled fraction) of the plasmid DNA. Increasing the plasmid yield has been well studied, but there is minimal information about increasing the supercoiling of the plasmid. A study carried out by Adamcik et al. highlighted how temperature affects the distribution of topoisomers in plasmid preparation and the impact on supercoiling of the preparation [9]. For instance, it has been reported that by achieving a high cell density culture, the plasmid yield is improved as plasmid production is directly linked to the biomass yield of a culture [10]. Some of these reports have achieved high biomass yield by engineering the cell using gene knockout or gene overexpression. Examples include the knockout of the *pts* gene responsible for glucose transport and replacement with *galp+* which codes for permease [11,12], modification of pentose phosphate pathway [13], and knocking out the pyruvate kinase gene [14].

In addition, attempts have been made to modify culture media to achieve high plasmid yield. O’Kennedy et al. [15] reported that at a specific ratio of carbon to nitrogen, the plasmid yield is increased. Supplementation of optimized concentrations of glutamate and ammonium chloride have also been reported to improve plasmid DNA production [16]. It has also been reported that a semi-defined medium supplemented with casamino acid (SDCAS) supported high yield and plasmid stability as compared with a medium containing soy amino acids and LB medium [15]. All of these reports have tackled the plasmid yield, however, maintaining a high supercoiling is still a challenge that needs addressing.

In addition to a high supercoiled amount being required for the effectiveness of plasmid use for vaccine and gene therapy applications, it makes plasmid more compact as the plasmid becomes coiled due to the twisting and writhing associated with supercoiling. This is potentially essential for downstream processing of plasmid as these molecules are sensitive to shear during downstream processes such as centrifugation, flow through pumps [17,18], and filtration. The sensitivity of plasmid DNA to shear is also dependent on the size of the plasmid, hence by making plasmid more compact we reduce the sensitivity on exposure to shear.

Several studies have investigated external factors and their effects on plasmid supercoiling [19,20,21]. However, there are very few reports that have investigated the effect of plasmid DNA modification aimed at increasing supercoiling and superhelical density (measurement of the compactness of the DNA) of the plasmid DNA [22]. The DNA negative supercoiling is introduced by the DNA gyrase, a Type II topoisomerase that is tightly regulated alongside the DNA topoisomerase I, which relaxes the DNA [23]. DNA gyrase overexpression in *E. coli* has only led to a slight increase in plasmid supercoiling [24]. 

The catalytic activity of the DNA gyrase involves binding to a specific sequence on the DNA and using energy from ATP to introduce a negative supercoil into the DNA [25]. Identifying the sequence and exploiting the extent binding increases the rate at which gyrase introduces supercoils into the DNA. A number of strong gyrase binding sites have been identified in plasmid pBR322, pSC101, and the bacteriophage Mu. The Mu-phage gyrase binding sequence has been reported to have a strong affinity for the binding of gyrase, hence stimulating the replication and packaging of the Mu bacteriophage in its host [26]. This sequence is found in the middle of the 37 kb genome of the bacteriophage Mu and contains an 8 bp sequence where the gyrase binds and inserts supercoil into the Mu genome [27]. 

Exploiting the efficiency of the Mu-phage SGS for increasing DNA supercoil and superhelical density means increasing the supercoil nature of the plasmid further. Further studies have shown that the length of the left and right of the key sequence on Mu-phage SGS also affects the efficiency of the sequence to insert a supercoil into the plasmid DNA. In a separate study, Hassan et al. [22] tested different lengths of the SGS and found that the 398 bp sequence, which is the longest of the different SGS lengths tested, significantly increased the plasmid supercoiling and superhelical density of a 2.7 kb plasmid.

Several strains of *E. coli* have been selected for the production of plasmid DNA based on laboratory use and commercial availability. These include *E. coli* K-12 strains such as JM108 [28], DH5α [11,15,29], GALG20 [11], BL21(DE3) [30], SCS1-L [31], and DH10B [32]. Usually, host cells are selected based on experiments carried out on the various strains available. Yau et al. [19] demonstrated that there is no direct correlation between the strain and the plasmid, and therefore testing on different strains should be carried out when selecting a strain for specific plasmids.

In this study, we investigate the impact of the bacteriophage Mu strong gyrase binding site sequence engineered into a 6.8 kb plasmid and amplified in four different *E. coli* strains which have been selected based on past studies, as well as to examine the effect of the potential increase in supercoiling on the integrity of the plasmid during downstream processing. 

## 2. Materials and Methods

### 2.1. Strains amd Plasmids 

The pSVβ-galactosidase plasmid vector was employed as the parent plasmid. The plasmid contained the gene coding for ampicillin resistance which was used for its selection upon transformation with a suitable host. The plasmid also contained the SV40 early promoter and enhancer sequence that drove the transcription of a specific *lacZ* gene on the plasmid. The plasmid was purchased from Promega Corporation (Southampton, UK). The Mu-phage SGS (strong gyrase-binding site) sequence used in this project was as reported by Hassan et al. [22]. The 398 bp sequence was selected from among the three sizes that were reported in the study. The selection of the sequence as a strong binding site for the DNA gyrase in *E. coli* was based on the report by Pato and Banerjee [26]. The Mu-phage SGS sequence was taken from the European Nucleotide Archive of the European Bioinformatics Institute (EBI). The sequence was found on position 17,681 bp–18,078 bp of the 37 kb bacteriophage Mu genome. The sequence was prepared as a synthetic gene and cloned into the plasmid vector by Genscript Corporation (Genscript, Picastaway, NJ, USA). The resulting plasmid vector was named pSVβ-gal398.

The *E. coli* strains selected for this study were based on the studies of Yau et al. [19]. The strains were also selected based on their industrial relevance and commercial availability. Several strains were derivatives of the parent *E. coli* strain K-12. Table 1 shows the summary of the genotype of these strains. All of the strains that were acquired in-house or commercially purchased, were then transformed with plasmids pSVβ-Gal and pSVβ-Gal398 using a standard heat shock calcium chloride technique [33]. The cells were prepared as glycerol stocks and were transferred into cryovials and stored at −80 °C until required.

The transformed cells, stored in glycerol, were thawed and used to streak LB agar (yeast extract, 5.0 g/L, tryptone 10.0 g/L, sodium chloride 5.0 g/L, and agar 15 g/L) plates which contained ampicillin for the selection and growth of plasmid containing cells. The ampicillin concentration used was 100 µg/mL. The plates were then incubated at 37 °C for 24 h, wrapped in film, and stored at 4 °C until they were required.

### 2.2. Fermentation

Two cultures (5 mL and 10 mL) containing LB media and the appropriate antibiotics (ampicillin) were grown in 50 mL falcon tubes for 16 h at 37 °C in an incubator with an agitation speed of 250 rpm. The media were inoculated with single picked colonies from freshly prepared agar plates. For shake flask fermentation, the inoculum was prepared by inoculating 10 mL of culture from glycerol stock and incubating it in a shaker at 37 °C overnight. The overnight culture was used to inoculate 200 mL of LB media containing ampicillin in a 1 L shake flask and placed in an orbital shaker at 37 °C with an agitation speed of 250 rpm. For the 5 mL culture, samples were taken from the culture at the end of the fermentation and the optical density (OD_600nm_) was measured with a spectrophotometer at a wavelength of 600 nm. The OD was used to predict the biomass yield per mL of culture. 

For the shake flask culture, samples were taken from the culture hourly and the optical density was measured using a spectrophotometer with a wavelength of 600 nm. The OD was used to predict the biomass yield per mL of culture. The optical density obtained was used to plot the growth curve of the fermentation.

### 2.3. Plasmid Extraction and Purification 

Plasmid DNA samples from the fermentation was extracted and purified using the Qiagen Spin Mini Prep kits and the Qiagen Maxi prep kits (Qiagen Ltd, Sussex, UK). The procedure was followed according to the protocols provided with the kits by the manufacturer. The plasmid yield was determined by measuring the concentration of the plasmid DNA in the purified samples. The concentration was determined by spectrophotometry using the Nanodrop ND-1000 Spectrophotometer (Nanodrop, Willington, DE, USA). 

### 2.4. Determination of Plasmid Topology

To determine the different topoisomers (linearized, open-circular, and supercoiled) of the plasmid DNA present in the samples, linearized and open circular (also known as nicked) plasmid samples were prepared and run alongside the samples during agarose gel electrophoresis. To prepare open-circular plasmid, 2 µL of the enzyme *Nt.Bst.NBI* (NEB, UK) was added to a preparation of 2 µL plasmid DNA, 5 µL of 10X buffer, and 41 µL of water. The mixture was incubated at 55 °C for 1 h followed by inactivation of the enzyme at 80 °C for 20 min. To prepare the linear plasmid, 2 µL of the enzyme *BamHI* (NEB, UK) was added to the preparation of 2 µL plasmid DNA (equivalent to 1 µg), 5 µL of 10X Cutsmart buffer, and 41 µL of water. The mixture was incubated at 37 °C for 15 min.

The plasmid DNA topology compositions and percentage intensities were determined using agarose gel electrophoresis and 0.8% (*w/v*) agarose gel was prepared in 1X TAE (tris-acetate-EDTA) buffer (Sigma-Aldrich, St. Louis, MI, USA). The gel was prestained with ethidium bromide (500 µg/mL) and run in 1X TAE (tris-acetate-EDTA) buffer at 90 V for 2 h. The gel was viewed under a UV light and the image was acquired using the AlphaImager Mini System (ProteinSimple, San Jose, CA, USA). A subsequent method involved the use of SYBR^®^ Gold which was used to stain the gel after electrophoresis. The stained gel was viewed under blue fluorescence using the Amersham Imager 600 (GE Healthcare). The AlphaView CA Software (ProteinSimple, Version 3.4.0.0, San Jose, CA, USA) was used to identify the bands and as a densitometry tool to quantify the intensity of the bands on the acquired image of the gel.

### 2.5. Determination of Plasmid Superhelical Density

The plasmid super-helical density was determined using chloroquine agarose gel electrophoresis with a slight modification of the method reported by Bowater [39]. Agarose (0.7% *w/v*) was prepared in 1 × TBE (tris-borate-EDTA) buffer (Sigma Aldrich). Before running, 1.2 mg/L of chloroquine diphosphate was used to prestain the gel. The sample was loaded, and electrophoresis was carried out in the first dimension at 2 V/cm for 20 h with 1 × TBE containing the same concentration of chloroquine diphosphate as the running buffer. The electrophoresis was stopped, and the gel was allowed to soak in 1 × TBE buffer with 6 mg/L chloroquine diphosphate for 3 h. Electrophoresis was carried out in the second dimension, 90° to the first at 1.8 V/cm for 24 h with 1 × TBE containing the same concentration of chloroquine diphosphate in the soaking buffer as the running buffer. Gel was then rinsed three times in water for an hour each. The gel was stained with SYBR gold nucleic acid stain for 1 h before visualization under a UV light. The superhelical density was calculated as follows:

(1) $$\sigma =\frac{\Delta Lk}{Lk_{o}}$$

(2) $$Lk_{o}=\frac{N_{o}}{h_{o}}$$

where, *ΔLk* is the linking difference determined from the gel image as the number of bands, *Lk_o_* is the linking number determined from the size of the plasmid in bp, *N*, and number of base pairs per each helical turn assumed to be 10.5 bp.

### 2.6. Plasmid Shear Exposure Experiment

To carry out the shear experiments, the plasmids were diluted in TE buffer to a concentration of 15–20 µg/mL. The ultra-scale down (USD) shear device was set to the maximum shear rate of 18,000 rpm (equivalent to 10^6^ s^−1^). Then, 20 mL of the sample was injected into the USD shear device while avoiding bubbles. With the exception of the controls, the sample was exposed to shear for 20 s, as exposing the sample beyond this time had no effect on the plasmids [40]. After exposure to shear, samples were collected and run on agarose gel electrophoresis to determine the percentage of supercoil content. The supercoiled content was expressed as a fraction of the supercoiled content of the control.

### 2.7. Plasmid Hydrodynamic Diameter

The plasmid hydrodynamic diameter (D_h_) was determined using the nanoparticle tracking analysis (NTA). This was done using the Nanosight NS300 (Malvern Instrument, UK). Using an inlet syringe pump, 1 mL of sample prepared in filtered TE buffer was injected into the sample chamber. The laser module was then mounted, and the camera started. Once the recording was complete, standard measurement tracking analysis was carried out in triplicate and the average recorded on the NTA software. The output showed the distribution of particles in the sample with mean size and modal size recorded. A detailed protocol is reported in [41].

## 3. Results

### 3.1. Effects of SGS on Plasmid Yield and Supercoiling and Strain Selection

**Figure 1:**
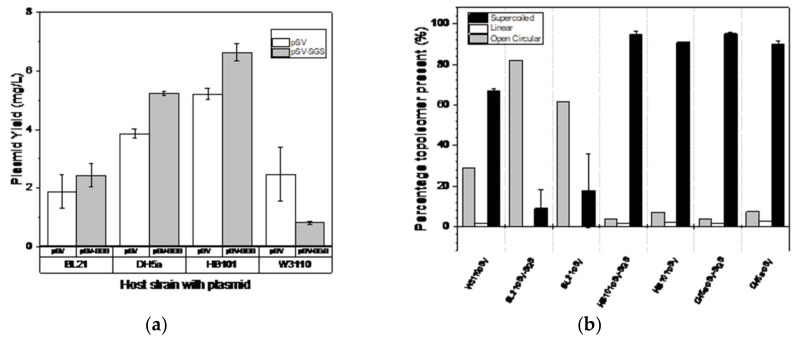
Plasmid yield and supercoiling of pSVβ-gal398 (pSV-SGS) and pSVβ-gal (pSV) amplified in the four strains. All analyses were done in triplicates. (**a**) The total plasmid yield (mg/L) of pSVβ-gal398 and pSVβ-gal amplified in the four strains and (**b**) percentage supercoiled topoisomer content of plasmid extracted from the four strains used. The differences observed were tested statistically with *p* < 0.05.

Figure 1 shows the results observed for the four different strains selected with respect to the plasmid yield and supercoil topoisomer content of the extracted plasmid. A similar fermentation approach was implemented on the four strains as detailed in the materials and methods. Results are the average of the triplicates for each strain-plasmid combination. The biomass yield is reported as OD600nm. All results were subjected to statistical analysis to test for significantly different observations. A significantly higher plasmid yield was observed for SGS-containing plasmid pSVβ-gal398 as compared with the parent plasmid pSVβ-gal. The increase plasmid yield for pSVβ-gal398 was observed in the strains DH5α and HB101 with an increase of 36% and 27%, respectively, as compared with pSVβ-gal (*p* < 0.05 when subjected to a t-test between the two plasmids in each strain, however this was not the case for the other two strains W3110 and BL21(DE3). 

The supercoiled content was determined according to the method describe in the materials and method section. The supercoil percentage shown in the figure is expressed as mean of triplicates ± SEM the differences observed was subjected to a paired t-test with a confidence limit of 95%. Similar to the plasmid yield discussed above, a high supercoiled content was observed in DH5α and HB101 with supercoiled content for both SGS-containing plasmid and non-SGS plasmid exceeding 90% as shown in Figure 1B. However, there was a statistically significant increase (5%) in the supercoiled content for pSVβ-gal398 as compared with the parent plasmid pSVβ-gal (*p*-value < 0.05 in both strains).

**Figure 2:**
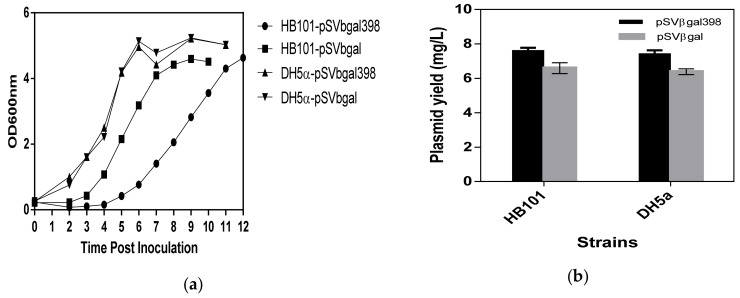
Characteristics of shake flask fermentation of the two strains HB101 and DH5α. Charts show (**a**) growth curve of the strains bearing both the non-SGS plasmid and SGS containing plasmid and (**b**) plasmid yield of pSVβ-gal398 and pSVβ-gal amplified in the two strains. Results are the mean of triplicate samples (n = 3).

Figure 2A shows results of the shake flask fermentation growth curve for *E. coli* strains DH5α and HB101 harboring pSVβ-gal398 and pSVβ-gal. Fermentations were run in triplicates. Figure 2B shows a plot of plasmid yield for *E. coli* strains DH5α and HB101 harboring pSVβ-gal398 and pSVβ-gal. The differences observed between the two plasmids for each strain are statistically significant as it was subjected to a t-test with a confidence limit of 95% (*p* < 0.05). The plasmid yield differences observed between the two strains were not statistically significant when subjected to a statistical test (*p* > 0.05). 

### 3.2. Effect of SGS Presence on the Superhelical Density of the Plasmid

**Figure 3:**
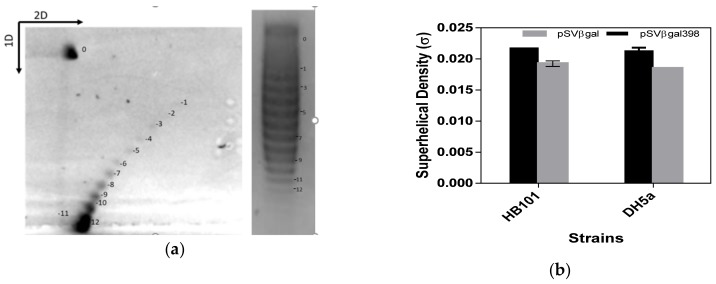
Superhelical density determination of plasmid amplified in DH5α and HB101. (**a**) 2D and 1D chloroquine-agarose gel electrophoresis showing the distribution of topoisomers of the plasmid. The bands depict the linking difference which is then used to calculate the superhelical density (**b**) Chart showing the superhelical density of pSVβ-gal398 and pSVβ-gal amplified in the two strains. N = 4. The difference observed is statistically significant in the two strains (*p* < 0.05).

Figure 3a shows a DH5α sample two-dimensional and one-dimensional chloroquine agarose gel which was run to determine the linking difference of the plasmid. Each sample was run on a single gel, hence it is impossible to show all sample gels. Each band represents the different level of supercoil that is found on the plasmid. The linking difference with the linking number was used to calculate the superhelical density of the plasmid according to the equations in materials and methods. The result for triplicate samples was taken. Figure 3B shows the result of the comparison of the superhelical density of the two plasmids (pSVβ-gal398 and pSVβ-gal) amplified in the two strains HB101 and DH5α expressed as mean ±SEM. As shown in the graph, the superhelical density of pSVβ-gal398 was approximately −0.022 in both strains as compared with −0.019 for pSVβ-gal (with *p* < 0.05).

### 3.3. Impact of Increase Supercoiling on Plasmid Integrity

**Figure 4:**
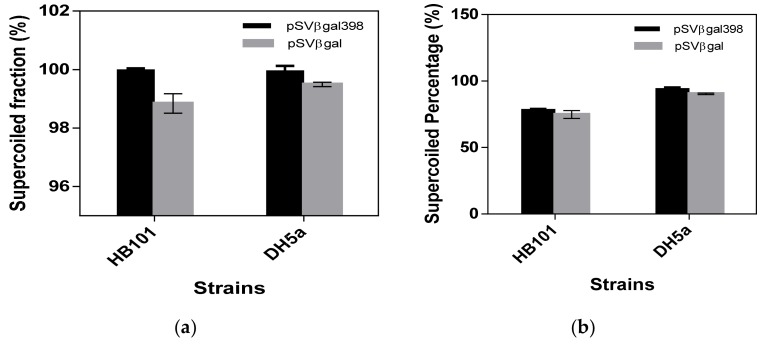

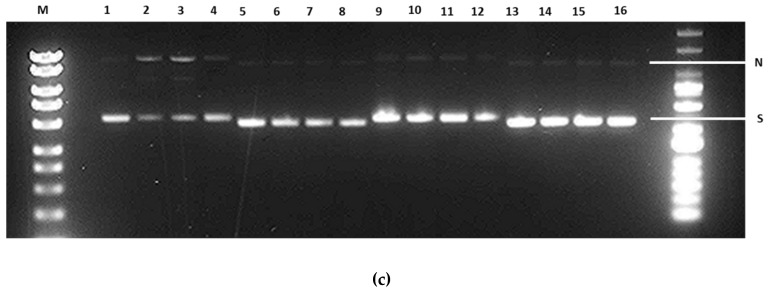
(**a**) Supercoiled topoisomer content after exposure to shear using the USD shear device, expressed as a fraction of the control (supercoiled content of non-sheared sample), n = 3 (**b**) Supercoiled content of the control sample before shearing. (**c**) Gel image of the samples after shearing. M = marker, Lane 1 = DH5α/pSVβ-gal398 control sample, Lane 2–4 = DH5α/pSVβ-gal398 sheared samples, Lane 5 = DH5α/pSVβ-gal control sample, Lane 6–8 = DH5α/pSVβ-gal sheared samples, Lane 9 = HB101/pSVβ-gal398 control sample, Lane 10–12 = HB101/pSVβ-gal398 sheared samples, Lane 13= HB101/pSVβ-gal control sample, Lane 14–16 = HB101/pSVβ-gal sheared samples, N = nicked plasmid, and S = supercoiled plasmid.

The plasmid integrity was determined by measuring the supercoiled content of plasmid preparation when exposed to shear. Details of the experiment are in the materials and method section. The shear stress is expressed as the energy dissipation rate and the supercoiled fraction was determined. Figure 4 shows the plot of supercoiled fractions for the two plasmids in each strain. The supercoiled fraction was determined by comparing the supercoiled content of the sheared plasmid relative to the control (non-sheared sample) and expressed as a percentage of the control. Supercoiled fractions are reported as mean ± SEM. Despite both plasmids maintaining a supercoiled fraction above 90%, there was a higher supercoiled fraction in the plasmid bearing SGS. The differences observed between the plasmid for each strain are statistically significant with a *p*-value <0.05 and a confidence limit of 95%. This observation is not surprising as it has been reported that plasmid <13 kb are affected minimally by shearing during downstream processing with significant shear observed for plasmid larger than 13 kb [17,18]. Figure 4c shows the gel image of the sheared plasmid and quantification carried out by densitometric method. Most of the sheared plasmid end up as nicked topology as labelled in the figure.

### 3.4. Determination of Plasmid Compactness by Particle Hydrodynamic Diameter Measurement

**Figure 5:**
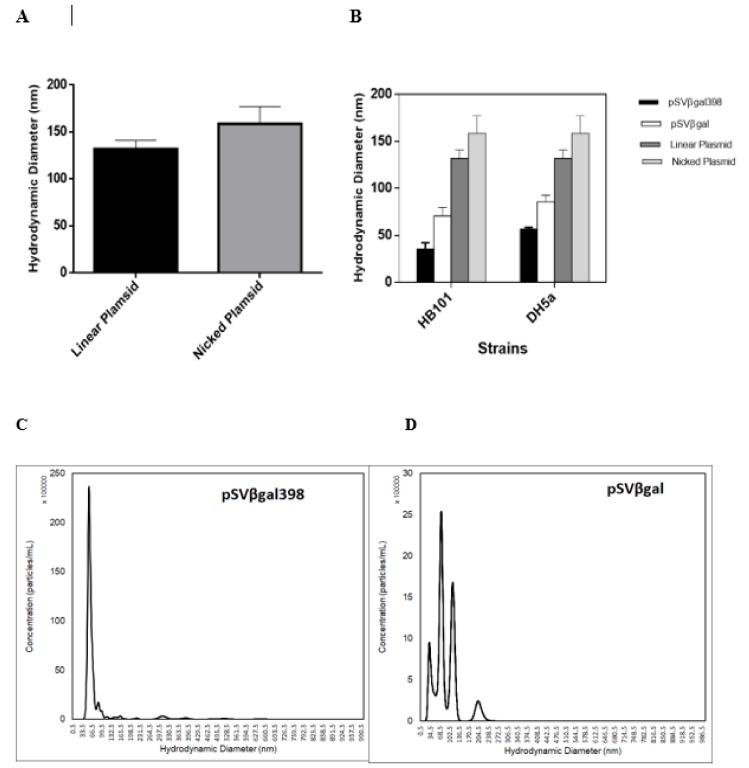
Particle size determination using the nanoparticle tracking analysis which tracks particle based on Brownian motion. Charts showing (**A**) the hydrodynamic diameter (*D_h_*) of linearized and nicked pSVβ-gal398 plasmid and (**B**) the hydrodynamic diameter of supercoiled plasmid pSVβ-gal398 and pSVβ-gal in the two strains. The sizes are compared to the nicked and linearized plasmid which are larger than the supercoiled form of the plasmid. (**C**) and (**D**) show the particle size distribution of the plasmid pSVβ-gal398 and pSVβ-gal from the strain DH5α. It shows the plot of diameter against concentration in particles/mL. The analyses were done in triplicate for all samples.

Figure 5 shows the results of plasmid hydrodynamic diameter determination using the nanoparticle tracking analysis (NTA) in an attempt to measure the determine plasmid compactness by size measurement. The NTA measures polydisperse particles and Figure 5c,d show the distribution of plasmid in a sample preparation of pSVβ-gal398 and pSVβ-gal amplified in HB101. The peaks are the concentration (particles/mL) of the different isoform of plasmid present in the preparation. The mode of the distribution was used in the analysis as it was expected that most of the plasmid particles were in the supercoiled form. It was confirmed from the densitometry method that the majority of the plasmids were in the supercoiled form. In order to confirm this from the NTA analysis, the hydrodynamic diameter of the nicked and linear plasmid was measured for comparison. Figure 5a shows the hydrodynamic diameter of linearized and nicked pSVβ-gal398. The hydrodynamic diameter is expressed as mean ± SEM of the triplicate samples. Figure 5b shows a plot of the sizes of pSVβ-gal398, pSVβ-gal linear, and nicked plasmid amplified in strains HB101 and DH5α. The results are mean ±SEM of the triplicate samples. The summary of the hydrodynamic diameter is shown in Table 2. The result shows that pSVβ-gal398 is more compact with a lower hydrodynamic diameter as compared with pSVβ-gal in both strains used.

## 4. Discussion

As a result of the progress in some clinical trials involving DNA vaccines, it is expected that these DNA therapeutics will be ready for human use in the next few years [5,6,7]. This means that a cost-effective production platform needs to be ready for mass production. A cost-effective platform would include a high yield and high supercoiled plasmid producing process. Supercoiling is essential for the application of DNA as vaccines and for gene therapy applications. DNA supercoiling is important for processes such as replication, transcription [42], as well as unknotting and decatenation of double stranded DN which are both required for efficient DNA metabolism [43]. Studies have investigated the impact of supercoiling on the efficacy of vaccine with one report suggesting a plasmid preparation containing above 70% supercoiled topoisomer is capable of inducing immunity compared to the open circular topoisomer and linear topoisomer [8]. In addition, the FDA recommends that plasmid preparation should contain >80% supercoiled fraction, and the expectation of the bioprocess industry is that plasmid preparation should contain >90% supercoiled content [44]. Hence, it is important to focus on increasing both the yield and the supercoiled topoisomer of plasmid preparation to meet the expected production of therapeutic DNA products.

We have previously identified that a 398 bp Mu-phage SGS induces supercoiling of plasmid DNA [22]. In this study, the 398 bp Mu-phage SGS sequence was introduced into a 7.2 kb plasmid pSVβ-gal. The study was carried out in small-scale with different strains of *E. coli* transformed with the SGS and non-SGS containing plasmids. The aim of investigating different strains was to observe the dependency of Mu-SGS on the host strain and to select the best-behaving strain(s). The selected strains were based on the previous report by Yau et al. [19] who reported that plasmid amplification and optimization is host-strain dependent. Therefore, we studied how much impact the host strain would have on the amplification of the recombined plasmid.

There was a high plasmid yield observed in two of the strains used (HB101 and DH5α) for plasmid bearing SGS (pSVβ-gal398). The high plasmid yield observed in DH5α agrees with the review of Lara and Ramirez [10], highlighting DH5α as a high plasmid yield producing strain specifically designed for plasmid production. HB101 on the other hand has not been extensively employed for plasmid production, most likely due to its high carbohydrate contents which may cause problems during downstream processing [45], however there are a few studies who have reported high plasmid yield using this strain of *E. coli* [19,46]. The significance of the increase in plasmid yield is implicated in the overall plasmid yield which is reported to be around 65% for a plant designed to produced 141 g per batch [47].

The supercoiled topoisomer content was also considered for strain-dependence amplification of the SGS-containing plasmid, as this is one of the bioprocess requirement for plasmid DNA production for therapeutic application [48]. Similar to the plasmid yield discussed above, a high supercoiled content was observed in DH5α and HB101, with supercoiled content for both SGS-containing plasmid and non-SGS plasmid exceeding 90%, as shown in Figure 1B. However, there was a statistically significant increase (5%) in the supercoiled content for pSVβ-gal398 as compared with the parent plasmid pSVβ-gal (*p*-value < 0.05 in both strains). This shows the impact of the presence of the Mu-phage SGS sequence which has been reported to increase the supercoiling of DNA due to strong binding of the gyrase [22,49]. The binding of the gyrase to the SGS site induces a nick on the plasmid and enhances supercoiling of the plasmid [24,50,51]. The enhancement of supercoiling is shown by the increased superhelical density of the plasmid which is discussed later. A large proportion of the preparation in BL21(DE3) was nicked, as shown in Figure 1B. This shows again that the effect of SGS presence on the supercoiling of the plasmid DNA is strain dependent. A similar result was observed for W3110. 

Therefore, on the basis of the result of the initial screening studies on the strains, we selected the two best-behaving strains for further study. The increase in the amount of supercoiled plasmid in the preparation coupled with the increase in plasmid yield further led to an increase in the overall productivity with a 27% for pSVβ-gal398 as compared with the unmodified pSVβ-gal.

Figure 2 shows the results of the shake flask fermentation growth curve for *E. coli* strains DH5α and HB101 harbouring pSVβ-gal398 and pSVβ-gal. The final biomass yield observed agreed with initial studies with no significant differences between host strain harbouring pSVβ-gal398 and strain harbouring pSVβ-gal. We did not expect that there would be a significant difference although the presence of the extra SGS sequence in the pSVβ-gal398 could have added to the metabolic stress on the strain [52], but since it was not expressing any additional recombinant product, the effect would be minimal. However, there was a lower growth rate observed in the strain HB101 but not in DH5α. The presence of the extra sequence on the plasmid may have led to the reduced growth rate observed particularly in HB101 [53]. In addition, the differences in the genotype of both strains may have played a role in the differences observed in the growth curve, for example, DH5α contained a *gyrA96* mutation in the gyrase gene [54]. This mutation may have played a role in the differences observed. The fact that the decrease in growth rate was observed only in HB101 reiterates the reports that bioprocessing of plasmid DNA is strain dependent [19]. 

The plasmid yield was observed to be higher for pSVβ-gal398 in both the strains 7.63 mg/L and 7.44 mg/L in HB101 and DH5α, respectively as compared with the parent plasmid pSVβ-gal with a yield of 6.59 mg/L and 6.38 mg/L in HB101 and DH5α, respectively. The increase in plasmid yield was as a result of an increase in plasmid copy number (not shown). The plasmid copy number was investigated as it was clear that the observed increase in plasmid yield was not due to an increase in biomass yield, as the final biomass was similar for the strain-plasmid combinations for the two *E. coli* strains investigated. The increase in copy number may have been influenced by the presence of SGS on the plasmid, however, this needs further investigation. Another possible reason for the increase in plasmid yield, specifically in HB101, may be the low growth rate, because a low growth rate has been reported to support plasmid amplification [48]. When comparing the two strains carrying the modified plasmid, the differences in the plasmid yield observed may again be as a result of the *gyrA96* mutation in DH5α which is not present in HB101.

Having established the significance of SGS on the yield and supercoiling of plasmid, we set out to determine the extent of supercoiling by determining the superhelical density (σ) of the plasmid. This is a measure of how supercoiled a plasmid is in terms of the twist (that is the winding of the DNA helix around its axis) and writhe (the number of times the DNA helix crosses itself) of the two strands on one another [55]. The superhelical density of DNA also measures if the DNA is positively supercoiled or negatively supercoiled. This is determined using the linking number, which quantitatively describes the topology of the plasmid in terms of twist and writhe. With the exception of extreme thermophiles [51], negatively supercoiled DNA is the most preferred topological form of DNA as it facilitates the interaction between replication/transcriptional proteins and DNA [56]. As shown in Figure 3, the superhelical density of pSVβ-gal398 was approximately –0.022 in both strains as compared with –0.019 for pSVβ-gal (*p* < 0.05). This agrees with earlier report by Hassan et al [22] who observed the same result in a 2.8 kb plasmid. However, as this was a small-sized plasmid, it was necessary to investigate if this effect was observed for a medium-sized plasmid, as plasmids for therapeutic applications are likely to be in the range of >6 kb

The increase in superhelicity or superhelical density observed in plasmid carrying the SGS sequence translates to high negative supercoiling. The implication of the high superhelicity to bioprocessing challenges is the possibility of maintaining the integrity of the plasmid during downstream unit operations such as centrifugation and filtration as they would be more compact and less sensitive to shear [17,57]. Moreover, it has been reported that supercoiling is essential for efficient transfection [58]. Therefore, the increase in superhelicity of the plasmid could potentially increase the transfection efficiency of the plasmid [58,59,60]. The increase in superhelical density is equivalent to an average of two extra linking number differences (*ΔLk*) in pSVβ-gal398 compared to pSVβ-gal. However, both plasmids are negatively supercoiled.

One of the potential benefits of a highly supercoiled plasmid is the maintenance of plasmid integrity during downstream processing due to its high compactness [57]. Plasmid DNA molecules are exposed to elongational shear stress during the centrifugation process as well as in pumps [17,18]. Shear stress causes nicking of the plasmid hence reducing the supercoiled plasmid topoisomer content of the final plasmid preparation. We tested how pSVβ-gal398 behaves when exposed to shear since the higher superhelical density means it is more compact. The results showed that there was a reduction in the supercoiled content of pSVβ-gal as compared with pSVβ-gal398. The supercoiled content of plasmid pSVβ-gal398 remained intact despite exposing them to shear, although the supercoiled content of the two plasmids preparation was not less than 90%. This observation is not surprising as it has been reported that plasmid with a size of <13 kb are affected minimally by shearing during downstream processing with significant shear observed for plasmid larger than 13 kb [17,18]. The maintenance of integrity could be attributed to the increase in compactness of the plasmid [57]. 

An attempt was made to quantify the compactness of the plasmid by determining the hydrodynamic diameter of the plasmids. Conformational changes induced by SGS presence were determined by measuring the hydrodynamic diameter of the plasmid using nanoparticle tracking analysis (NTA). The NTA measures the hydrodynamic diameter based on the diffusion coefficient of the particle in Brownian motion and then tracks the particle to establish the diameter of the particle. It is able to track single particles with high resolution and detect both high and small particles simultaneously [61]. In addition, NTA is able to measure the concentration of particles in solution [62]. The plasmid hydrodynamic diameter (*D_h_*) was determined using the Nanosight NS300 (Malvern, UK) and both the plasmids pSVβ-gal and pSVβ-gal398 amplified in the strains HB101 and DH5α were examined. 

Figure 5 shows the average hydrodynamic diameter of the linear and nicked plasmid. It is expected that the nicked plasmid will be larger than the linear plasmid as it has been observed during gel electrophoresis that supercoiled plasmid will migrate the fastest due to its tight packing, followed by linear plasmid, with the nicked plasmid migrating the slowest. The supercoiled plasmid is, therefore, expected to have a smaller size relative to linear and nicked plasmid. The hydrodynamic diameter observed also highlighted the compactness of the pSVβ-gal398 as ccompared with pSVβ-gal. For plasmids amplified in the *E. coli* strain HB101, the modal hydrodynamic diameter for the pSVβ-gal398 is 40 nm while the modal hydrodynamic diameter of the pSVβ-gal is 70 nm (Figure 5B). This supports the observation from the two-dimensional chloroquine agarose gel electrophoresis, where the superhelical density of pSVβ-gal398 was found to be higher than pSVβ-gal. The same was observed for plasmids amplified in the *E. coli* strain DH5α where the modal hydrodynamic diameter of the pSVβ-gal398 and pSVβ-gal were observed to be 57 nm and 86 nm, respectively (Figure 5B). This observation agrees with result observed during the measurement of superhelical density where pSVβ-gal398 had a higher superchelical density and hence more compact. The compactness observed is attributed to the presence of the Mu-SGS on the plasmid as reported earlier. This study highlights the possibility of investigating plasmid DNA hydrodynamic size using the nanoparticle tracking analysis (NTA) and to further prove the influence of Mu-SGS presence on the compactness of the plasmid DNA which are requirements for DNA uses for therapeutic applications.

Surprisingly, the effect of Mu-SGS on plasmid supercoiling was observed to be more prominent in plasmids amplified in HB101 in comparison to DH5α, a common *E. coli* strain for plasmid production. Although, there was an impact of Mu-SGS on the plasmid in both strains, the superhelical density and plasmid hydrodynamic diameter (*D_h_*) show that the plasmids amplified in HB101 are more compact and supercoiled than plasmids amplified in DH5α (Table 2). The possible explanation goes back to the differences in genotype, and in particular the *gyrA96* mutation, as there is a direct interaction between DNA gyrase and the Mu-SGS. This mutation could be limiting the activity of the DNA gyrase on the Mu-SGS due to conformational changes induced by the mutation. This is also evident from the investigation of the effect of shear. As shown in Figure 4, pSVβ-gal398 amplified in HB101 were more resistant to shear effect assuming a 100% maintenance of integrity after exposure to shear. Yau et al. [19] reported a strain dependency on plasmid production. This study further emphasizes the selection of the right strain for specific plasmid during bioprocessing.

## 5. Conclusions

The results of this study show that the yield and extent of supercoiling in plasmid is improved by modifying the plasmid, as supercoiling is required for the application of plasmid as biotherapeutics. By increasing the supercoiling, the overall yield of supercoiled plasmid is increased significantly, hence, solving the bioprocess challenge of plasmid yield and quality. We also showed that the effect of SGS is not limited to a 2.8 kb plasmid as we reported earlier [22]. Increasing the supercoiling also increases the compactness of the plasmid. This is potentially beneficial during downstream processing as an increase in compactness reduces the nicking of plasmid caused by shear exposure. 

We also reported that the effect of Mu-SGS to improve the supercoiling of the plasmid DNA is strain dependent, since the effect was more pronounced in strain HB101 as compared with DH5α. Although DH5α has been widely adopted as a favourite for plasmid production, there is an opportunity for adopting HB101 as a choice of strain. The extent of supercoiling was quantified by measuring the superhelical density of the DNA, a term which employs the linking difference of the plasmid. 

To investigate the effect of the Mu-SGS presence on the overall compactness, the hydrodynamic diameter of the plasmid was measured using nanoparticle tracking analysis (NTA). This method was used over the dynamic light scattering (DLS) method because it employs a number-weighted measurement rather than an intensity-weighted average of the DLS. The number-weighted measurement is more reliable as the plasmid preparation is polydispersed. The supercoiled plasmid hydrodynamic diameter was compared to the linearized and nicked plasmids. The results from the NTA also confirmed that the Mu-SGS containing plasmid was more supercoiled compared to the parent plasmid, further strengthening the observations with the superhelical density determination. The study also highlights the possibility of quantifying the extent of plasmid supercoiling using the nanoparticle tracking analysis (NTA). 

## Figures and Tables

**Table 1:** Strains selected for the study and their genotype.

Strain	Genotype	Source	Status
**BL-21**	$F^{-}\;dcm\;hsdS\left(r_{B}^{-}m_{B}^{-}\right)gal\Delta \left(DE3\right)$	[34]	Commercial
**DH5α**	$F^{-}\;endA1\;glnV44\;thi\;-1\;recA1\;relA1\;gyrA96\;deoR\;nupG\;\Phi 80dlacZ\Delta M15\;\Delta \left(lacZYA\;-argF\right)U169,\;hsdR17\left(r_{K}^{-}m_{K}^{+}\right),\lambda^{-}$	[35,36]	Commercial
**HB101**	$F-mcrB\;mrr\;hsdS20\left(rB-mB-\right)recA13leuB6ara\;\;-14\;proA2\;lacY1\;galK2\;xyl-5\;mtl\;\;-1\;rpsL20\left(SmR\right)\;glnV44\;\lambda -$	[37]	Non-commercial
**W3110**	$F-\lambda -rph-1\;INV\left(rrnD,rrnE\right)$	[38]	Non-commercial

**Table 2:** Superhelical density and hydrodynamic diameter of the plasmids amplified in different strains. The hydrodynamic diameter is expressed as mean + SEM and n = 3.

	HB101		DH5α	
	pSVβ-gal398	pSVβ-gal	pSVβ-gal398	pSVβ-gal
**Superhelical density**	−0.0218	−0.0192	−0.0204	−0.0185
**Hydrodynamic diameter**	39.53 ± 6.52 nm	70.77 ± 8.84 nm	56.97 ± 1.55 nm	85.50 ± 7.19 nm
**Compactness**	44% more compact		34% more compact	
